# Meningiomas-Related Epilepsy After Surgery

**DOI:** 10.3390/cancers17091523

**Published:** 2025-04-30

**Authors:** Francesca Battista, Giulia Cultrera, Cristina Andreea Aldea, Eleonora Visocchi, Alberto Parenti, Giovanni Muscas, Camilla Bonaudo, Davide Gadda, Riccardo Carrai, Antonello Grippo, Alessandro Della Puppa

**Affiliations:** 1 Department of Neurosurgery, Careggi University Hospital, 50134 Florence, Italy; 2 Medical and Surgical Department, Department of Neurofarba, University of Florence, 50134 Florence, Italy; 3 Department of Neuroradiology, Careggi University Hospital, 50134 Florence, Italy; 4 Department of Neurophysiopatology, Careggi University Hospital, 50134 Florence, Italy

**Keywords:** meningioma, epilepsy, antiseizure medications, peritumoral edema

## Abstract

Background: Meningioma-related epilepsy (MRE) is observed in approximately 30% of patients. Although studies focus on identifying risk factors related to pre- and postoperative MRE, there is no clear evidence regarding the timing for discontinuing antiseizure medications (ASMs) after surgical resection. Methods: We retrospectively collected data from a series of naïve supratentorial meningiomas treated with surgical resection. Preoperative MRI was used to calculate the meningioma and peritumoral edema (PE) volumes through a voxel-based system. We analyzed the frequency of pre- and postoperative epilepsy in the group of meningiomas with and without perilesional edema (with PE > 1 cm^3^ as the cut-off). Results: From a clinical series of 507 patients, we included 128 who underwent surgical resection in our center between January 2020 and December 2022, with a mean follow-up of 30.1 ± 19.8 months. Surgical treatment had a curative effect on MRE (41.4% preoperative vs. 19.5% postoperative; *p* = 0.0001). We observed a statistically significant reduction in the seizure rate in cases with preoperative PE (45.3% preoperative vs. 18.9% postoperative; *p* = 0.0002) and a non-statistically significant reduction in cases without PE (32.5% preoperative vs. 21.4% postoperative; *p* = 0.24). We observed ASM continuation in 37.8% of Engel IA patients. Conclusions: PE increases the likelihood of MRE resolution with surgery. Our results show that surgical resection directly impacts MRE and ASM discontinuation in the presence of preoperative PE. The PE is a reassuring factor in decision-making regarding the timing of ASM discontinuation after surgery.

## Simple Summary

There is limited evidence regarding the epileptogenesis and management of meningioma-related epilepsy (MRE). Our study aims to identify risk factors for postoperative MRE to guide clinicians in discontinuing antiseizure medications (ASMs) after treating supratentorial meningiomas. Our results show that preoperative peritumoral edema (PE) is the only factor that can predict an excellent seizure outcome following meningioma resection, likely because surgery leads to its resolution. Furthermore, PE is also associated with a higher risk of preoperative epilepsy, highlighting its potential key role in the epileptogenesis of MRE. Therefore, based on our clinical experience, preoperative PE may be a positive factor in the decision-making process for discontinuing ASMs after surgery. To our knowledge, this finding has not been previously reported in the literature.

## 1. Introduction

Meningioma-related epilepsy (MRE) occurs in 30% of patients with supratentorial meningiomas and is, in 20–50% of cases, the presenting symptom [1,2]. Surgical resection leads to recovery in 60% of cases, with persistence of MRE after surgery in 30–40% of cases [3]. MRE significantly impacts patients’ quality of life due to recurrent seizures, which leads to limitations in daily activities’ autonomy and the side effects of antiseizure medications (ASM), primarily affecting cognitive functions [4,5]. Although the mechanisms of epileptogenesis in MRE are not fully understood, the initiation of ASMs is recommended after the first epileptic seizure in the presence of a supratentorial meningioma [6]. Therefore, the indication for ASM therapy is independent of the tumor’s radiological characteristics, seizure semiology, and, in general, patient-specific factors. This highlights how the definition of MRE encompasses a wide variety of patients who must start ASM independently of each character.

While the presence of a supratentorial meningioma may justify such a broad indication for ASM initiation, the same cannot be said for the continuation and discontinuation of ASMs throughout the clinical course of these patients. There is no recognized protocol for discontinuing ASMs after radical resection of a supratentorial meningioma. Due to a lack of clear scientific evidence, the decision of when and whether to stop these medications remains entirely at the clinician’s discretion, and this is one of the main unresolved issues in clinical practice regarding managing MRE [7]. Although numerous clinical studies have identified risk factors for MRE [8,9,10,11,12,13], none have pinpointed risk factors that could support a recognized protocol for postoperative ASM management. Clinicians often choose to continue ASMs for many years after radical meningioma surgery, even in the absence of seizures, due to the fear of recurrent seizures. In light of this, we decided to investigate which characteristics of epileptogenic naïve meningiomas in the preoperative period could reassure clinicians in their decision to discontinue ASMs.

## 2. Materials and Methods

Our study retrospectively collected a series of patients with naïve supratentorial meningioma surgically treated at our center between January 2020 and December 2022. The retrospective data collection was carried out using our hospital’s computerized database. We included all patients with naïve supratentorial meningioma who underwent radical surgical resection, provided that preoperative and follow-up clinical and radiological data were available for at least 12 months.

We excluded cases of recurrence/post-surgical residuals, WHO grade III or grade II cases that underwent adjuvant therapy, multiple meningiomatosis, death within 12 months after treatment, and all cases in which preoperative and postoperative MRI and clinical data were unavailable.

**Figure 1:**
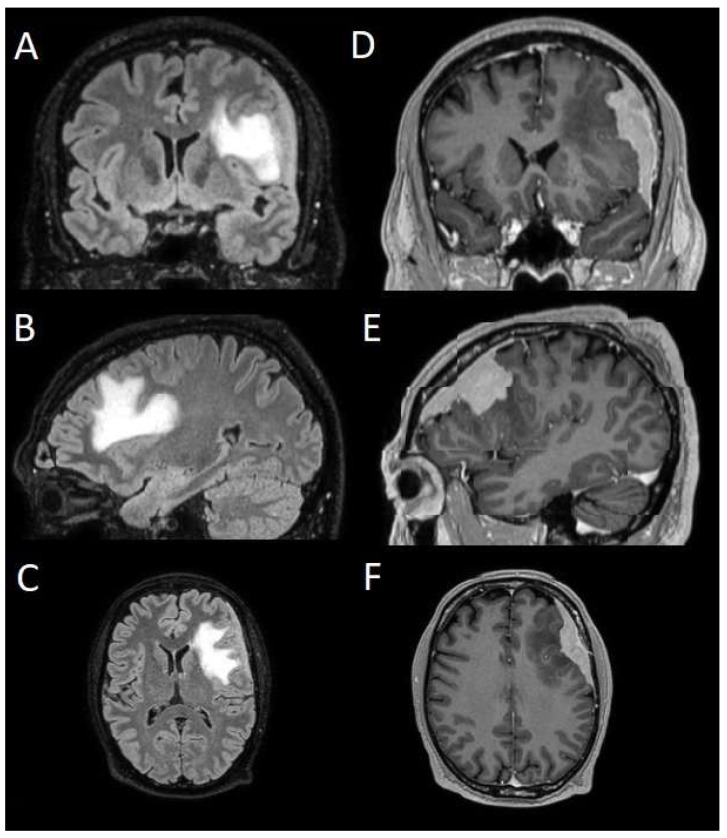
Sequences selected for the segmentation. (**A**–**C**) Coronal, sagittal, and axial FLAIR sequences to quantify PE. (**D**–**F**) Coronal, sagittal, and axial T1-weighted sequences with contrast enhancement to estimate meningioma volume. FLAIR: fluid-attenuated inversion recovery; PE: peritumoral edema.

**Figure 2:**
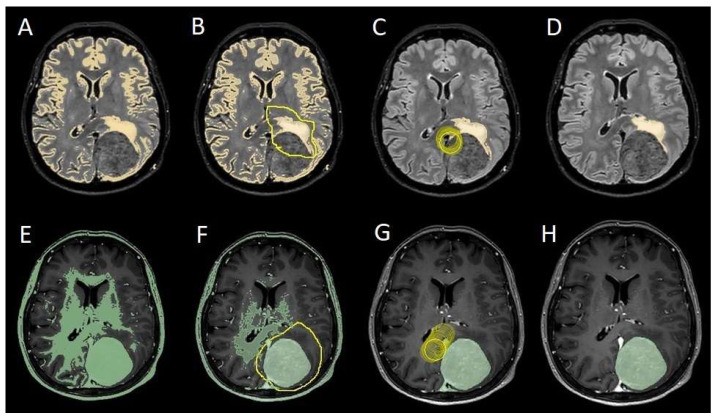
Example of segmentation process with the Slices website. (**A**,**E**) Automatic thresholding technique to identify initial regions of interest based on intensity values, respectively, for the PE on the FLAIR sequence and the meningioma on the T1-weighted-with-gadolinium sequence. (**B**,**F**) Partially automatic erasure of redundant signal with “erase outside” tool. (**C**,**G**) Definition of boundaries of edema and tumor through manual erasure. (**D**,**H**) Final segmented volumes. FLARI: fluid-attenuated inversion recovery; PE: peritumoral edema.

The patients underwent magnetic resonance imaging (MRI) at our center using a 1.5T or 3T Tesla MRI machine (Ingenia 3T, Philips Medical Systems, Best, The Netherlands) with the standard oncological protocol [14]. Specifically for analysis, the sequences collected were T1-weighted with contrast enhancement to estimate tumor volume and conformation and FLAIR (fluid-attenuated inversion recovery) to quantify PE (Figure 1). We manage the preoperative imaging in a DICOM (digital imaging and communications in medicine) format. The images from the T1-weighted sequences with contrast enhancement and FLAIR sequences in the DICOM format were processed through the Slicer website [15]. Tumor and PE segmentation was performed using a voxel-based analysis that integrated automated and manual methods (Figure 2).

**Figure 3:**
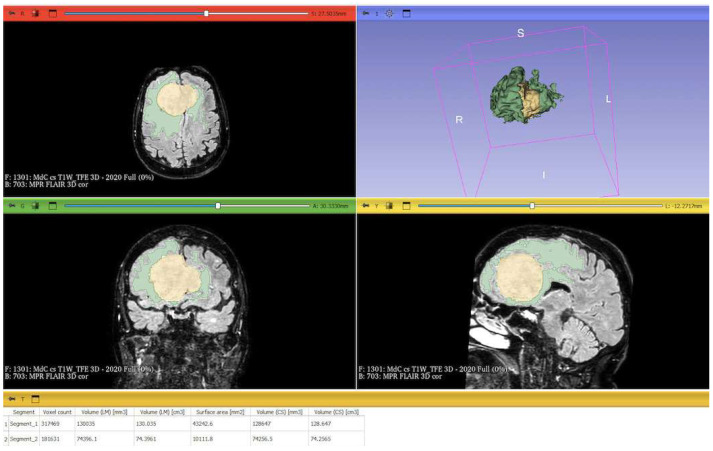
Screenshot of 3D Slicer showing the completed segmentation of the meningioma and PE, with 3D reconstruction and statistical quantification: the segmented PE is shown in green; the segmented meningioma is shown in yellow; and at the bottom, the statistical quantification of the segment volumes is displayed. The MRIs include a T1-weighted sequence and a FLAIR sequences merged at 50%. PE: peritumoral edema; MRI: magnetic resonance image; FLARI: fluid-attenuated inversion recovery.

**Figure 4:**
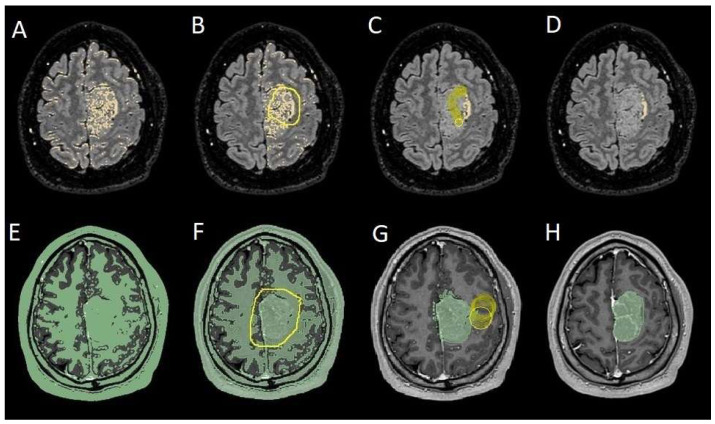
Segmentation process for PE < 1 cm^3^. (**A**,**E**) Automatic thresholding technique to identify initial regions of interest based on intensity values, respectively, for the PE and the meningioma. (**B**,**F**) Partially automatic erase of redundant signal with “erase outside” tool. (**C**) Precisely definition of boundaries of edema through manual erasure, cutting all around redundant signal. (**G**) Manual erasing boundaries for tumor volume. (**D**,**H**) Final segmented volumes. PE: peritumoral edema.

The process begins with an automatic thresholding technique to identify initial regions of interest based on intensity values (Figure 2A,E). This is followed by manual refinement to enhance accuracy and delineate precise boundaries (Figure 2B,C,F,G). The final segmentation provides volumetric measurements for both the tumor and the PE, aiding in quantitative analysis (Figure 3). The included patients’ pre- and post-treatment clinical data were retrospectively extracted from our center’s computerized database. Collected data included demographic information, preoperative clinical details (presence or absence of epilepsy, onset symptoms, ASM therapy and number of ASMs taken, and radiological characteristics of the meningioma), and postoperative data (Engel class [16], persistence or discontinuation of ASMs, ASM discontinuation timing, and possible postoperative functional deficits). A single examiner conducted data collection to minimize subjective variability in assessments. Surgical procedures were performed using a transcranial approach under general anesthesia. We excluded cases of surgical resection performed via an endoscopic endonasal approach. Cases of postoperative death (within one year after surgery) were excluded. The extent of resection (EOR) was determined based on the postoperative MRI (usually one month after surgery) and classified according to the Simpson grading system [17]. This study included cases of complete macroscopic meningioma resection (Simpson I, II, and III). Cases with residual tumor persistence or recurrence after surgery (Simpson IV and V) were excluded (Figure 4).

The postoperative seizure outcome was assessed using the Engel classification [16] one year after surgery, and we then updated the data to the most recent follow-up available at the time of inclusion in this study. We considered the most recent follow-up time to assess the Engel class.

Measurement quantitative data were expressed as mean ± standard deviation (SD). We analyzed qualitative variables by summarizing them as frequencies and percentages, and relationships between variables were assessed using Fisher’s exact test and the chi-square test. Analysis of variance (ANOVA) or the “N-1” chi-squared test was used to assess statistical differences between the two groups or percentages. A *p*-value < 0.05 was considered statistically significant. ORs and RRs were calculated to assess the statistical significance of associations. ORs were used to measure the strength of association between categorical variables, while RRs were calculated to estimate the risk of an event in one group compared to another. These methods were chosen to accurately assess the significance of relationships between the analyzed variables.

**Figure 5:**
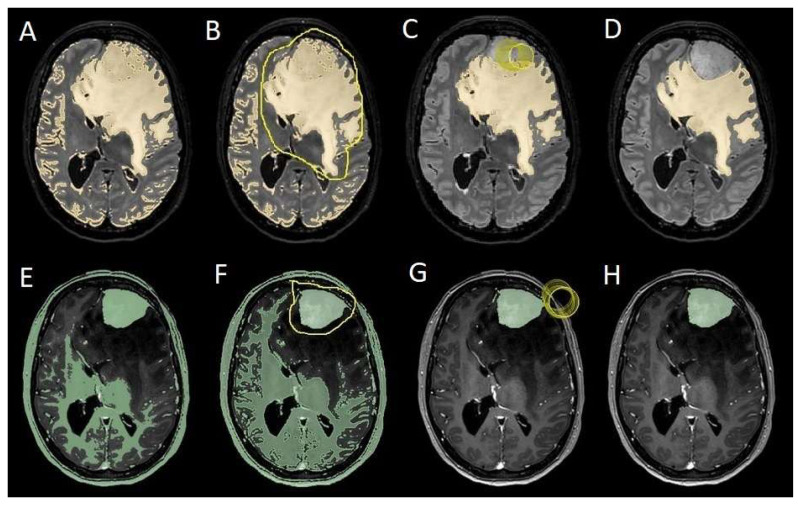
Segmentation process for PE > 1 cm^3^. (**A**,**E**) Automatic thresholding technique to identify initial regions of interest based on intensity values, respectively, for the PE and the meningioma. (**B**,**F**) Partially automatic erase of redundant signal with “erase outside” tool. (**C**) Precisely definition of boundaries of edema through manual erasure, cutting all around the redundant signal inside the tumor. (**G**) Manual erasing boundaries for tumor volume. (**D**,**H**) Final segmented volumes. PE: peritumoral edema.

To evaluate which independent variables (meningioma volume, PE volume, the ratio between meningioma and PE volume, and the presence of preoperative epilepsy) influenced the dependent variable (seizure outcome), we performed a binomial logistic regression, which required converting continuous variables into binary values. For PE volume, values < 1 cm^3^ were coded as 0 (Figure 4), and values ≥ 1 cm^3^ as 1 (Figure 5). The cut-off was set at 3 cm^3^ for tumor volume, and for the volume ratio, the cut-off was 1. The absence of preoperative epilepsy was coded as 0, while its presence was coded as 1. Regarding seizure outcome, Engel IA cases were coded as 0, and cases classified as Engel > IA were coded as 1.

To evaluate the effect of meningioma location on preoperative epilepsy and seizure outcome, we conducted two multivariate logistic regressions. The dependent variable was “preoperative epilepsy” in one model and “seizure outcome” in the other; the latter was defined using the Engel classification (Engel IA: 0; Engel > IA: 1). The independent categorical variables included “convexity”, “sphenoidal wings”, “falx cerebri”, and “olfactory grooves”. Both regressions used “olfactory grooves” as the reference category.

We conducted a binary logistic regression in the subpopulation of patients with preoperative epilepsy to evaluate whether any characteristics of epilepsy (seizure semiology, frequency, initiation of ASM therapy) could influence seizure outcome, coded as 0 for Engel IA and 1 for Engel > 1A. Among the variables, we considered seizure semiology as focal or primarily generalized and frequency as greater or less than one seizure per month.

We assessed the factors influencing the discontinuation of ASMs in the postoperative period using generalized estimating equations (GEE) for whom we had available follow-up data of at least 24 months. We considered “ID patient” as a subject variable and “timing” (i.e., the status of ASM therapy at the 12-month and 24-month follow-up time points) as a within-subject variable. Using an exchangeable correlation structure, we included the Engel class, the timing of follow-up, PE, and the interaction between the Engel class and the follow-up timing as a covariate.

The statistical analysis was conducted using IBM SPSS Software (IBM Corp. Released 2023. IBM SPSS Statistics for Windows, Version 29.0.2.0, Armonk, NY, USA: IBM Corp).

## 3. Results

**Figure 6:**
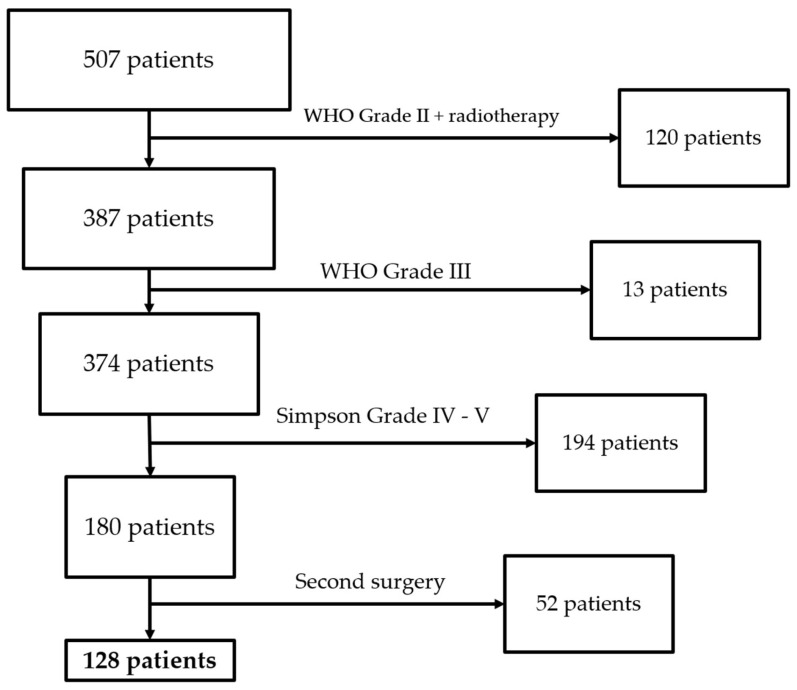
Patients flow diagram.

We collected 507 patients at our center between January 2020 and December 2022. We excluded 120 patients because they attained WHO grade II with adjuvant radiotherapy, 13 because they attained WHO grade III, 194 because they attained a Simpson grade of IV–V, and 52 because they were second surgery cases. We then included 128 patients in the study who underwent surgical resection (Figure 6). The demographic characteristics of the population are reported in Table 1.

All meningiomas included were naïve. In 64 cases, the meningioma was located along the convexity (50%); in 27 cases (21.1%), it was located along the sphenoidal wing; in 29 cases (22.7%), it was located at the level of the falx cerebri; and in 8 cases (6.2%) it was located in the olfactory grooves. The WHO grade was I in 105 cases (82%) and II in 13 (18%). The Simpson grade I–II was achieved in 110 patients (85.9%), and grade III–IV was achieved in 18 patients (14.1%). In all cases of WHO grade II, the Simpson grade was I or II. The mean follow-up period was 30.1 ± 19.8 months. We included 43 males (33.6%) and 85 females (66.4%), with a mean age of 64 years and a median of 65 years. Preoperative epilepsy was recorded in 53 cases (41.4%). During clinical follow-up, Engel IA was observed in 103 patients (80.4%). The ASM therapy was kept in 39 out of 103 patients with Engel IA (37.8%) and 19 out of 25 patients with Engel > IA (76%). Among patients on ASM therapy preoperatively who were classified as Engel IA postoperatively (39 patients), 14 discontinued ASM during follow-up (35.9%).

In the population of patients with preoperative epilepsy (53 cases), we observed focal seizures in 16 cases (30.2%) and primarily generalized seizures in 37 (69.8%). At least one ASM was used in 45 cases (84.9%), and seizure frequency was greater than once per month in 22 cases (41.5%).

ASM therapy was ongoing preoperatively in 58 cases (45.3%). At 12 months of follow-up, ASM therapy was maintained in 59 patients (46.1%). At 24 months of follow-up, ASM therapy was maintained in 38 patients (29.7%). A meningioma volume greater than 3 cm^2^ was observed in 122 cases (95.3%), and the presence of PE was recorded in 85 cases (66.4%), while a volume ratio greater than one was observed in 51 cases (39.8%).

### 3.1. Binomial Logistic Regression

**Figure 7:**
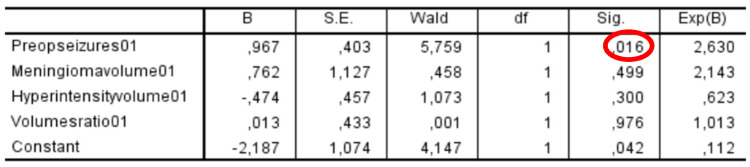
Binomial logistic regression: only preoperative seizure significantly correlated to the dependent variable “seizure outcome” with *p* = 0.016. The other independent variables analyzed (the volume of meningioma, the volume of PE, and the volume ratio) are not statistically correlated to the seizure outcome. PE: peritumoral edema.

A binomial logistic regression analysis was conducted to determine the independent variables associated with developing postoperative epilepsy. Among the variables analyzed, the only factor that showed a statistically significant association with postoperative epilepsy was the presence of preoperative epilepsy (OR = 2.88; 95% CI: 0.86–5.05; *p* = 0.016), indicating that patients with epilepsy before surgery had a higher likelihood of experiencing seizures after the procedure. In contrast, other potential predictors, including meningioma volume, PE volume, and volume ratio, did not demonstrate a statistically significant correlation with postoperative epilepsy. These findings suggest that while tumor-related volumetric factors were not significant predictors in this cohort, preoperative epilepsy remains a key determinant of postoperative seizure occurrence (Figure 7).

**Figure 8:**
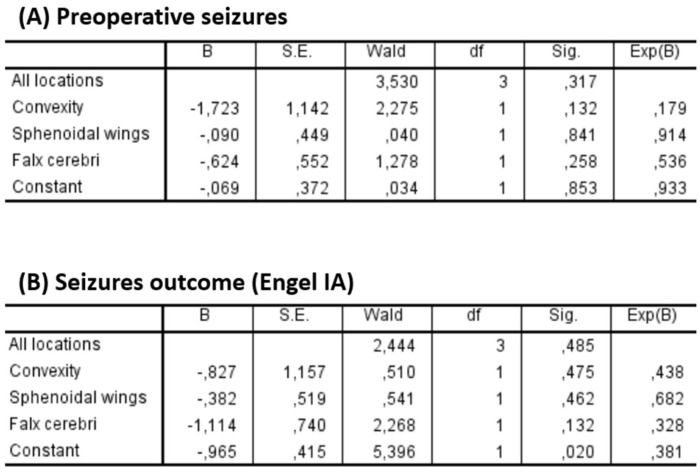
Binary logistic regression analysis of tumor location and its association with (**A**) preoperative seizures and (**B**) seizure outcome (Engel IA). None of the tumor locations (convexity, sphenoidal wings, falx cerebri) showed a statistically significant association with either preoperative seizure presence (*p* = 0.317) or seizure outcome (*p* = 0.485). While convexity meningiomas showed a trend toward lower odds of preoperative seizures (OR = 0.179; *p* = 0.132), the results were not statistically significant.

Two multivariate logistic regression models were performed to assess the impact of tumor location on epilepsy. The first model analyzed the association between location and preoperative seizures, while the second focused on seizure outcome. In both models, the olfactory groove was used as the reference category (Figure 8).

Regarding preoperative seizures, none of the locations showed a statistically significant association (*p* = 0.317 for the overall model). Convexity meningiomas showed a trend toward lower odds of preoperative seizures (OR = 0.179; *p* = 0.132), although this was not statistically significant.

Similarly, no significant association was found between tumor location and seizure outcome (*p* = 0.485 for the overall model). Falx cerebri lesions showed a non-significant trend toward poorer seizure outcomes (OR = 0.328; *p* = 0.132).

**Figure 9:**
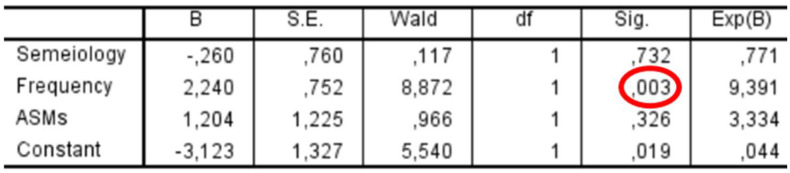
Binary logistic regression analysis in the subpopulation of patients with preoperative epilepsy. Among the variables included (seizure semiology, seizure frequency, and use of antiseizure medications), only seizure frequency > 1/month was significantly associated with worse seizure outcome (Engel > IA), with an odds ratio of 9.39 (*p* = 0.003). Seizure semiology and ASM use did not show statistically significant associations.

In the subgroup of patients with preoperative epilepsy (*n* = 53), a binary logistic regression was performed to assess whether seizure characteristics were associated with postoperative seizure outcomes (Figure 9). Among the predictors included—seizure semiology (focal vs. primarily generalized), seizure frequency (>1/month vs. ≤1/month), and use of ASMs—only seizure frequency showed a statistically significant association with outcome, with an odds ratio of 9.39 (*p* = 0.003). Seizure semiology (*p* = 0.732) and ASM use (*p* = 0.326) were not significantly associated with outcome.

### 3.2. Comparison of Proportion

**Figure 10:**
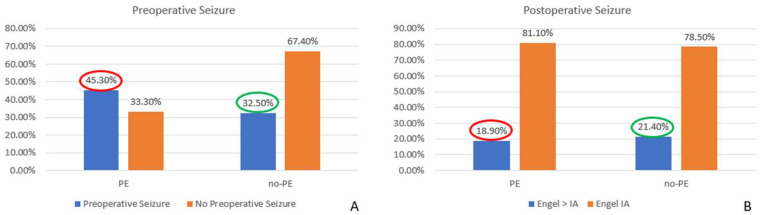
Comparison of proportion of the rate of pre- and postoperative seizures in the presence or absence of PE in the surgical group: the surgery significantly decreases the seizure rate in the presence of PE (45.3% vs. 18.5%; *p* = 0.0002), and does not in the absence of PE (32.5% vs. 21.4%; *p* = 0.24). PE: peritumoral edema.

When considering the overall impact of surgery on MRE, we found a significant decrease in seizure occurrence, with rates dropping from 41.4% before surgery to 19.5% postoperatively (*p* = 0.0001). These results highlight the potential curative effect of surgical resection in patients with MRE. Furthermore, we observed a statistically significant reduction in the seizure rate among patients with preoperative PE, decreasing from 45.3% before surgery to 18.9% postoperatively (*p* = 0.0002). This finding suggests that surgical intervention substantially impacted seizure control in this subgroup. Conversely, in cases without PE, although a reduction in seizure frequency was also observed (from 32.5% preoperatively to 21.4% postoperatively), this change did not reach statistical significance (*p* = 0.24), indicating that factors other than PE might influence seizure persistence in this cohort (Figure 10).

### 3.3. Generalized Estimating Equations

**Figure 11:**
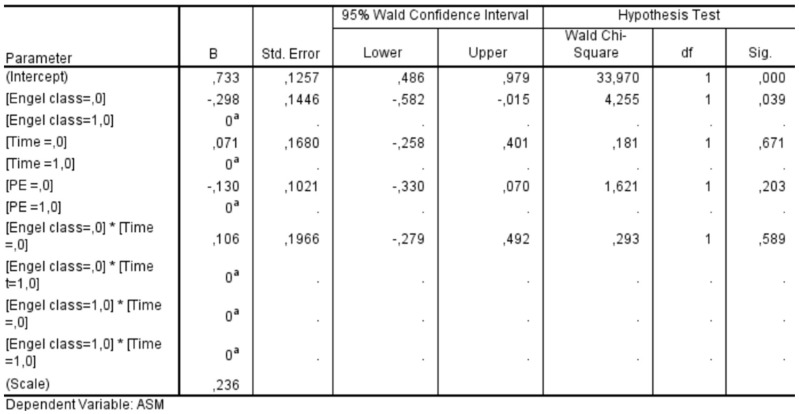
Results of the generalized estimating equations show how the Engel class influences the decision to discontinue or not to discontinue the ASM in the postoperative period, while the timing and the PE do not. ASM: antiseizure medication; PE: peritumoral edema. Notes: ^a^: Set to zero because this parameter is redundant.

We conducted a GEE analysis in a subgroup of 59 patients who had completed a 24-month follow-up period. This analysis allowed us to assess the influence of various factors on the likelihood of discontinuing ASMs after surgery. The statistical significance values obtained for each factor were as follows: Engel class demonstrated a significant association with ASM discontinuation (*p* = 0.039), whereas neither the timing of follow-up (*p* = 0.671) nor the presence of PE (*p* = 0.203) showed a statistically significant effect. Additionally, the interaction between Engel class and follow-up timing was insignificant (*p* = 0.589) (Figure 11). These findings indicate that Engel classification—a well-established measure of postoperative seizure outcomes—was the only independent factor significantly influencing the decision to discontinue ASMs in the postoperative period. In contrast, the timing of follow-up evaluations and the presence of PE did not play a decisive role in determining whether patients were taken off ASM therapy. This underscores the importance of seizure outcomes in guiding postoperative management strategies for epilepsy patients.

## 4. Discussion

Our study shows how the presence of PE is the only predictive factor for the optimal seizure outcome of the surgical resection of naïve supratentorial meningiomas and how its presence on preoperative MRI can be considered a factor in favor of ASM post-surgical discontinuation in the absence of seizures. Our study further highlights that a seizure frequency greater than once per month is a risk factor for a worse seizure outcome. Our work was conducted on a population of treatment-naive WHO grade I or II meningiomas, not subjected to adjuvant therapies after surgery and treated with surgical resection classified as Simpson grade I, II, or III. Our conclusions cannot be applied to meningiomas of WHO grade III or grade II that underwent postoperative radiotherapy.

The MRE is a widely studied topic in the literature, with the primary objective being the identification of risk factors for both preoperative and postoperative epilepsy. Despite many studies having been conducted, only a few risk factors have been commonly accepted as associated with MRE. In particular, preoperative epilepsy appears to be correlated with the presence of PE [10,18], while the only commonly accepted risk factor for postoperative epilepsy is the presence of seizures in the preoperative phase. A recent meta-analysis by Tanti et al. [19] reviewed more than 50 papers on supratentorial meningiomas and confirmed that PE is associated with an increased risk of preoperative epilepsy. Additionally, this meta-analysis suggested that PE might also be linked to a slight increase in the risk of both early and late postoperative epilepsy. However, the authors themselves acknowledged that PE does not appear to be the primary risk factor for postoperative seizures. Also, another recent meta-analysis confirms that PE is a predictive factor for postoperative late seizures [20]. These findings contradict our results, indicating that PE is a predictive factor for better seizure outcomes through surgical resection. The reasons behind the differing results can be attributed to several factors. First and foremost, both cited meta-analyses do not focus on the Simpson grade achieved or the WHO grade of the included studies. Postoperative epilepsy can be caused by residual tumor tissue or recurrence, introducing a bias in the assessment. In our case series, more than 85% of patients underwent resection with Simpson grade I–II, and the last 14.9% underwent Simpson grade III, and no macroscopic residual tumors were present postoperatively. Additionally, all the WHO grade III and recurrence cases during the follow-up period were excluded from our study. Consequently, postoperative epilepsy is attributable to factors independent of tumor residue/recurrence, thereby eliminating this evaluation bias. As a result, according to our binomial logistic regression, PE is not a risk factor for an Engel > IA outcome, meaning it is not associated with postoperative epilepsy. Another key difference between our study and previous ones lies in the approach to analysis. Having ruled out PE as a direct risk factor for postoperative epilepsy, we assessed whether its presence could influence the effectiveness of surgery in seizure control. Our findings indicate that in the presence of PE, surgery is more effective in controlling postoperative epilepsy, and to our knowledge, this is the first piece of evidence presented in the literature to date. Therefore, according to our analysis, the presence of PE is confirmed as a risk factor for preoperative epilepsy, but its resolution is also a predictor of excellent seizure outcomes following surgical resection. Surgery effectively resolves the mass effect of the meningioma on the surrounding parenchyma and venous system, leading to a reduction in PE itself [21,22,23]. If the presence of PE correlates with epilepsy, and if its resolution through surgery appears to favor a better seizure outcome compared to cases of surgical resection without PE, this raises the question of PE’s role in the epileptogenesis of MRE. There are few studies on this topic, and ours is the first that underlines the role of surgical PE resolution in controlling the seizure. However, our analysis shows that PE appears to be a significant factor that may play a key role in MRE epileptogenesis. Our results have two main implications: resolving PE is the key to treating the most common forms of MRE; second, when dealing with meningioma patients with PE, we must remember that resective surgery directly impacts PE resolution and, consequently, seizure freedom.

Another key finding of our study is that preoperative epilepsy increases the risk of a worse seizure outcome after surgery, with this risk being particularly elevated in patients experiencing more than one seizure per month. While the association between preoperative epilepsy and poorer outcomes has been previously reported [10], there is less evidence specifically linking higher preoperative seizure frequency to less favorable postoperative seizure control. This is a relevant result that may serve as a helpful element in predicting surgical seizure outcomes, and it could also be considered when making decisions regarding ASM discontinuation after surgery.

Currently, ASMs in patients with meningioma are indicated only in cases where epileptic seizures occurred before surgery [6]. There are no recommendations for their use as prophylaxis in the absence of seizures. The ASMs are often associated with side effects such as drowsiness, psychomotor slowing, and potentially severe drug interactions [24,25,26]. Consequently, chronic ASM therapy significantly reduces patients’ quality of life and should be avoided in unnecessary cases. However, even if the indication when starting ASM in MRE is reported, the evidence of the proper timing of ASM discontinuation after surgery is less clear. The fear of seizures and the absence of evidence has led neurosurgeons over the years to maintain the ASMs for excessively long periods or even never to discontinue them in patients of Engel class IA [27,28], and our clinical experience reflects this approach. In our surgical series, the decision to discontinue ASM was based on the seizure outcome rather than the duration of follow-up or preoperative PE presence. According to our study’s retrospective nature, we described our center’s past approach and reported an ASM maintenance rate of 37.8% in Engel IA cases. This rate is consistent with those reported in the literature [29], showing that more than one-third of seizure-free patients remain on ASM therapy. The recent literature trend of identifying preoperative risk factors for the persistence of postoperative epilepsy is aimed at preemptively determining who is the best candidate for postoperative discontinuation of ASM therapy, avoiding overtreatment [11].

The risk factors investigated in the development of postoperative epilepsy are numerous, and certain ones have been more consistently reported in the literature as being significantly associated with seizure persistence after surgery. Among these, factors such as the convexity location of the meningioma, involvement of the Rolandic regions, younger patient age, and larger meningioma volumes have been identified as relevant [8,30]. Despite the broad range of risk factors explored in different studies, most of those identified tend to be intrinsic characteristics of the meningioma or the patient, which, interestingly, do not necessarily appear to be directly related to the underlying mechanisms of MRE. Our study results indicate that PE is a suggestive factor for seizure resolution in the postoperative period, highlighting a potentially favorable prognostic implication for patients undergoing surgery and suggesting a potential role for PE in the epileptogenesis of MRE. This finding is particularly relevant in clinical decision-making as it provides a reassuring element when considering the discontinuation of ASM after surgery. Given that there are currently no well-defined guidelines or standardized protocols governing ASM withdrawal in this context, recognizing PE as a potentially favorable factor may offer additional confidence to clinicians in guiding postoperative management strategies. Although our findings represent a mere statistical correlation, and further studies are needed to explore the potential role of PE in the epileptogenesis of MRE, this result holds clinical significance. In patients with a history of epilepsy who remain seizure-free in the postoperative period, the presence of PE on preoperative MRI could become a key factor in deciding whether to initiate the decalage of ASM therapy.

Regarding the timing of the decalage, it is reasonable to hypothesize that it could coincide with the first postoperative MRI at follow-up in which PE is no longer detected. Based on these hypotheses, we suggest that a good protocol would be the following: in cases of WHO grade I meningiomas, and in Engel class IA at 3 months post-surgery, if no solid residuals are visible on follow-up MRI, and the previously present PE has disappeared, ASM decalage is advisable. Under the same conditions, but in the absence of PE on preoperative MRI or cases of persistent PE on postoperative MRI at 3 months, it is advisable to re-evaluate the case at 6 months post-surgery. In contrast, in cases of WHO grade II meningiomas, it is advisable to apply this protocol just in case no adjuvant therapy is needed due to reaching a Simpson grade of I, II, or III.

While further studies are required to confirm this hypothesis, the correlation between PE’s presence or absence and seizures’ occurrence or resolution could serve as a valuable clinical indicator for determining the optimal timing for ASM decalage and eventual discontinuation in the postoperative setting.

## 5. Conclusions

Our study highlights how PE is associated with a higher risk of preoperative MRE and how it can predict an excellent seizure outcome following the surgical resection of supratentorial meningiomas. The presence of preoperative PE should be considered a favorable factor for discontinuing ASM postoperatively in the absence of seizures. According to our results, the surgery directly impacts MRE and ASM discontinuation in the presence of preoperative PE.

## Figures and Tables

**Table 1:** Demographic and clinical data.

Variable	Details
Total patients included	128
Gender	
Male	43 (33.6%)
Female	85 (66.4%)
Age at diagnosis (y)	
Mean age	64
Median age	65
Mean follow-up (m)	30.1 ± 19.8
Location of meningiomas	
Convexity	64 (50%)
Sphenoidal wings	27 (21.1%)
Falx cerebri	29 (22.7%)
Olfactory grooves	8 (6.2%)
WHO grade	
Grade I	105 (82%)
Grade II	13 (18%)
Simpson grade (surgical group)	
Grade I and II	110 (85.9%)
Grade III	18 (14.1%)
Preoperative epilepsy	53 (41.4%)
Engel class IA	103 (80.4%)
Preoperative ASMs	58 (45.3%)
Postoperative ASMs	
12 months post-treatment	59 (46.1%)
24 months post-treatment	38 (29.7%)
Volumes	
Meningioma > 3 cm^3^	122 (95.3%)
PE	85 (66.4%)
Meningioma/PE > 1 cm^3^	51 (39.8%)

Notes: ASMs: antiseizure medications; PE: peritumoral edema.

## Data Availability

Data is unavailable due to privacy of the patients included.

## Abbreviations

The following abbreviations are used in this manuscript:

MRE	Meningioma-related epilepsy
PE	Peritumoral edema
ASM	Antiseizure medication
FLAIR	Fluid-attenuated inversion recovery
